# Sport Activity as Risk or Protective Factor in Feeding and Eating Disorder

**DOI:** 10.3390/bs9120143

**Published:** 2019-12-06

**Authors:** Salvatore Iuso, Antonello Bellomo, Tiziana Pagano, Raffaella Carnevale, Antonio Ventriglio, Annamaria Petito

**Affiliations:** Department of Clinical and Experimental Medicine, University of Foggia, 71122 Foggia, Italy; iuso.salvatore@libero.it (S.I.); antonello.bellomo@unifg.it (A.B.); tizianapaganops@gmail.com (T.P.); raffaella-carnevale@libero.it (R.C.); a.ventriglio@libero.it (A.V.)

**Keywords:** sport activity, protective factors, risk factors, self-esteem, mood states, eating disorders

## Abstract

Emerging evidence suggests controversial results on the associations between sport activity and eating disorders (EDs). The aim of the present study was to investigate the relationship between sport activity in general, weight-dependent/independent sport activity particularly, and risk or protective factors in feeding and eating disorder (FED). The sample (*n* = 282, divided into two successive groups), included competitive athletes in the first analysis, non-competitive athletes, and sedentary peers; in the second analysis it has been divided into weight-dependent athletes, weight-independent athletes, non-competitive athletes, and sedentary peers. The participants were tested with Rosenberg Self-Esteem Scale, Profile of Mood State (POMS) questionnaire, Body Shape Questionnaire (BSQ), Eating Attitudes Test (EAT-26), and Body Uneasiness Test (BUT). The results show higher levels of *self-esteem* among athletes in general and particularly in weight-independent athletes (*p* = 0.0210). We found higher levels of image and body dissatisfaction among sedentary peers and weight-dependent athletes (0.0005 < *p* < 0.0015). Sedentary peers also reported higher levels of tension/anxiety, depression/dejection, confusion/bewilderment and fatigue/inertia (0.0001 < *p* < 0.0331). Dieting and oral control were found to be higher among weight-dependent athletes (0.0337 < *p* < 0.0400). The findings suggest that *sedentary* condition is associated with higher levels of *body-image discomfort* and higher level of psychological distress, whereas weight-dependent athletes may report dietary issues and bodily concerns. Sport activity should be promoted and specific trainings on diet and body-consciousness encouraged among athletes.

## 1. Introduction

Pleasure activities, such as physical exercise and sport activity, should play a central role in a person’s well-being. Shields and Bredemeier (1995) affirmed that sport participation should influence personality, promoting courage, persistence, and self-control among athletes [1]. According to this assumption, sport psychologists investigated those dispositional factors such as personality traits, motivational orientations [2,3,4,5,6], and situational factors (e.g., levels of sport participation, types of sports, etc.) that could determine intra- and inter-individual differences in diminishing maladaptive behaviors in sport context, including eating disorders (EDs). Unfortunately, EDs appear to be insidious, and athletes are more at risk of EDs rather other young adults and the general population [7,8]. Negative ED outcomes include an increased risk of death [9,10] and other physical health problems [11], which are caused by three interplayed mechanisms, i.e., undereating, purging, and low body weight [12]. Increased fat mass during puberty worries teenage athletes in general and can influence the perception of oneself and the perception of sport performance among young athletes leading to them starting a diet [13,14,15,16,17,18,19]. Nattiv (2007) described the dangerous transition from healthy diet to a clinical disorder through a gradual continuum [20] that causes clinical manifestations, i.e., menstrual dysfunctions (amenorrhea) in females; reduced bone mineral density (osteoporosis); and disordered eating, including the restriction of food intake, selection of food groups, bingeing or purging behaviors, etc. [21,22]. EDs may also lead to multiple body consequences among athletes despite current high physical performance [23,24,25] and affect interpersonal functioning [26]. One group that has consistently been identified as being at higher risk of developing EDs is the *élite* athletes, which are involved in sports that are strongly based on weight and/or shape [18,19]. However, it is unclear whether athletes represent a subgroup that is truly “at risk” of experiencing an eating disorder [27]. In competitive athletes, some are more focused on “winning at all costs” (win-oriented) and this might lead to weight loss, increasing the risk of EDs. On the other hand, protective factors for EDs associated with sports, such as the promotion of persistence, personal satisfaction, good mood states, and good levels of self-esteem and self-control among athletes, could be underestimated [1]. A more focused research on risk and protective factors for EDs among athletes should be encouraged.

The purpose of this study was to determine the relationship between sport activity and risk factors (predisposition in favor of greater vulnerability) or protective factors (predisposition in favor of less vulnerability) in feeding and eating disorder (FED). We hypothesized that there could be different levels of self-esteem, self-control, mood states, eating attitudes, and body image among competitive athletes, non-competitive athletes, and sedentary peers. Furthermore, given existing evidence [28] that weight-dependent sports are risk factors for FED, we hypothesized that there could be different associations between both weight-dependent and weight-independent athletes with risk factors as well as protective factors for FED.

## 2. Materials and Methods

### 2.1. Participants

A total of 282 subjects (*n* = 122 female, *n* = 160 male), aged between 11 and 30 years, were recruited for participation in this study. The sample was recruited from sports centers of the Apulia Region, Italy (*n* = 14 swimming, *n* = 14 volleyball, *n* = 12 football, *n* = 13 rugby, *n* = 13 karate, *n* = 14 basketball, *n* = 14 dance), and from high schools (*n* = 94 non-competitive athletes, fitness and *n* = 94 sedentary peers). The sample was divided into two successive groups for two different analyses. In the first analysis it was divided into competitive athletes, non-competitive athletes (mostly composed of subjects practicing *fitness* about 3 h/week) and sedentary peers and in the second it was divided into weight-dependent athletes (swimming, volley, karate, dancing, and rugby), weight-independent athletes (football and basket), non-competitive athletes, and sedentary peers [15,16]. In weight-dependent athletes, we included those athletes that are more sensitive and vulnerable to changing their shape and body weight in order to improve sport performances. We considered rugby players as weight-dependent athletes on the basis of evidence that this group may show major weight concerns related to weight variability and discomfort with body-image [29].

Competitive athletes were those who competed, within the last year, at a regional or national level in their chosen sport (for example, swimming, volley, karate), certificated by CONI (Italian National Olympic Committee). All competitive athletes had been competing in championship competitions when the study was conducted, with at least 10 h of training/week.

### 2.2. Test

The measurements were based on demographic characteristics, anthropometrics, description of psychological traits, and clinical rating scales.

(a) Rosenberg Self-Esteem Scale [30,31] is a rating scale that is used both for research purposes and for measuring the effectiveness of prevention and health promotion interventions aimed at small groups. It consists of 10 items exploring global self-worth in terms of positive and negative feelings about the self.
(b) The Body Shape Questionnaire (BSQ) is a self-report questionnaire that was developed to measure concerns about body shape [32,33]. The maximum score is 204, and a higher score indicates more dissatisfaction and discomfort with the participant’s own body experience.
(c) Profile of Mood State (POMS) is a standard validated psychological test formulated by McNair et al. [34,35] consisting of 65 items that fit into six categories: Tension/anxiety (T/A), depression/dejection (D/D), anger/hostility (A/H), vigor/activity (V/A), confusion/bewilderment (C/B) and fatigue/inertia (F/I). The Profile of Mood States (POMS) has often been employed in studies measuring athletes’ emotional states.
(d) Eating Attitudes Test (EAT-26) [36,37] provides rates of eating attitudes on a six-point Likert scale, ranging from never to always, with higher scores suggesting greater ED symptomatology. EAT-26 total scores over 20 are considered to be indicative of a possible ED [24]. Internal consistency was good in the current sample (Cronbach’s alpha = 0.86). The EAT-26 items are grouped in three subscales: Dieting, bulimia and food preoccupation, oral control.
(e) Body Uneasiness Test (BUT) [38,39] is a self-assessment scale that explores dissatisfaction with the body and weight; avoidance and compulsive control; experiences of detachment and estrangement from one’s body; and specific concerns for certain parts, features, or bodily functions. The overall average score identifies the severity degree linked to one’s body image (GSI—global severity index). The sum of the scores of each group of items allows the investigation and evaluation of five smaller areas related to: Weight phobia (WP—weight phobia), concerns about body image (BIC—body image concerns), avoidance conduct (A—avoidance), compulsive controls of one’s own image (CSM—compulsive self-monitoring), depersonalization (D—depersonalization).

### 2.3. Procedures

The sample was recruited through the “Regional Project for the Prevention and Contrast of Eating Behavior Disorders promoted by the network of the Eating Disorder Centers of Apulia Region—Italy” organized by ARES (Regional Health Authority—Apulia) no. DG 1940/12 of 31/12. Regional Project objectives were:

- Description of feeding behaviors through the regional population;
- Evaluation of mood states, levels of self-esteem, and body perception among a group of athletes, non-competitive athletes, and sedentary peers;
- Clinical characterization of FED through the regional population.

The participants were informed of the aim of the study and completed informed consent as well as rating scales. Moreover, for subjects aged <18 years old, informed consent was obtained by parents. All data were managed to ensure confidentiality [40].

In addition, subjects did not receive any compensation either directly or indirectly from their participation in the research. The present study has been conducted in accordance with the Helsinki declaration and was approved by the local Institutional Review Board (Comitato Etico ASL-FG; prot. n. 09/CE/12).

The division of the sample involved in the study is presented in Table 1.

### 2.4. Statistical Analysis

Analysis were conducted using the statistical software Grand Prism 5 (San Diego, CA, USA). Means and standard deviation (SD) have been calculated for each studied parameter, and an alpha level of 0.05 was selected throughout the study. The differences in psychometric dimensions across the groups were compared using nonparametric Kruskal–Wallis test with Dunn’s multiple comparison post-hoc testing.

## 3. Results

The mean scores on sub-scales of the Rosenberg Self-Esteem, BSQ, POMS, EAT-26, and BUT for each studied group are presented in Table 2.

The analyses across the three groups (competitive athletes/non-competitive athletes/sedentary peers (CAs/NCAs/SPs)) showed a significant difference in *self-esteem* between CAs/NCAs vs. SPs (χ^2^ (3) = 8.07, *p* = 0.0177). There was no difference in the Rosenberg Self-Esteem score between the two groups of athletes (competitive vs. non-competitive).

The analyses on the sub-scales of BSQ among competitive athletes vs. non-competitive athletes vs. sedentary peers indicated higher levels of *self-image discomfort* in CAs > SPs > NCAs (χ^2^ (3) = 7.60, *p* = 0.0223), and higher scores in body image discomfort among CAs > SPs > NCAs (χ^2^ (3) = 7.40, *p* = 0.0248).

We employed the POMS for scoring emotional states among athletes and sedentary peers. There were significant associations in the following subscales: (a) higher levels of tension/anxiety (T/A) among SPs > CAs > NCAs (χ^2^ (3) = 7.70, *p* = 0.0213); (b) higher levels of depression/dejection (D/D) in SPs > NCAs > CAs (χ^2^ (3) = 11.54, *p* = 0.0031); (c) significantly higher vigor/activity (V/A) among CAs > SPs > NCAs (χ^2^ (3) = 21.57, *p* < 0.0001); (d) significantly higher levels of confusion/bewilderment (C/B) among SPs (χ^2^ (3) = 9.91, *p* = 0.0070) and (e) much higher scores of fatigue/inertia among SPs as expected (χ^2^ (3) = 18.61, *p* ˂ 0.0001).

The scoring at EAT-26 did not show any threshold condition for eating disorders through the sample even if we found higher levels of oral control (EAT-26) among NCAs and CAs > SPs (χ^2^ (3) = 6.30, *p* = 0.0428).

The analysis of BUT scoring and its subscale indicated greater body uneasiness (at GSI) in SPs than in the two groups of athletes (NCAs > CAs) (χ^2^ (3) = 6.36, *p* = 0.0416). In particular, SPs reported higher scores of body image concerns (χ^2^ (3) = 6.57, *p* = 0.0375) and compulsive self-monitoring (χ^2^ (3) = 6.64, *p* = 0.0362).

The mean scores on sub-scales of the Rosenberg Self-Esteem, BSQ, POMS, EAT-26, and BUT for four groups (weight-dependent athletes (WDAs), weight-independent athletes (WIAs), non-competitive athletes (NCAs), sedentary peers (SPs)) are presented in Table 3.

The analyses across the four groups indicated a significant difference in the self-esteem level, which is higher among athletes in general and particularly higher (24.12 ± 5.39) in WIAs (χ^2^ (4) = 9.73, *p* = 0.0210).

It is of note that we found higher levels of image and body dissatisfaction among SPs (30.71 ± 15.58 and 31.90 ± 16.12) and WDAs (31.90 ± 16.12 and 30.22 ± 13.40; *p* = 0.0005 and *p* = 0.0015).

There were no significant differences among WDAs and WIDs about mood states at POMS. There were significant associations in the following subscales: (a) higher levels of tension/anxiety (T/A) among SPs > WIAs > NCAs > WIAs (χ^2^ (4) = 8.73, *p* = 0.0331); (b) higher levels of depression/dejection (D/D) in SPs > NCAs > WIAs CAs > (χ^2^ (4) = 12.59, *p* = 0.0056); (c) significantly higher vigor/activity (V/A) among WDAs > WIAs > SPs > NCAs (χ^2^ (4) = 23.10, *p* < 0.0001); (d) significantly higher levels of confusion/bewilderment (C/B) among SPs (χ^2^ (4) = 11.18, *p* = 0.0108); and (e) much higher scores of fatigue/inertia among SPs as expected (χ^2^ (4) = 20.51, *p* ˂ 0.0001).

It is of note that we found higher levels of dieting in WDAs (3.33 ± 5.95; *p* = 0.0400).

The scoring at EAT-26 did not show any threshold condition for eating disorders through the sample even if we found higher levels of oral control (EAT-26) among WDAs (3.02 ± 3.64) and NCAs (2.83 ± 2.84), χ^2^ (4) = 8.69, *p* = 0.0337.

The analysis of BUT scoring and its subscale indicated greater body uneasiness (at GSI) among SPs and WDAs (χ^2^ (4) and = 23.08, *p* < 0.0001); in particular, we found higher scores of weight phobia in SPs and WDAs (1.29 ± 1.14 and 1.15 ± 0.92) (χ^2^ (4) = 19.63, *p* = 0.0002), body image concerns scored SPs > WDAs = NCAs > WIAs (χ^2^ (4) = 21.84, *p* < 0.0001), higher compulsive self-monitoring among SPs (χ^2^ (4) = 18.11, *p* = 0.0004) and higher depersonalization among SPs (χ^2^ (4) = 12.77, *p* = 0.0052).

## 4. Discussion

According to the epidemiological data of DSM 5 [41], FED most often occurs between the age of 10 and 30. The current study examined the relationship between sport activity and risk or protective factors for FED in subjects aged between 11 and 30 years.

Our results showed higher self-esteem scores in subjects practicing sport activity than sedentary ones. Moreover, the post-hoc analyses showed no differences in self-esteem between weight-dependent and weight-independent athletes. This may indicate that sport activity increases self-esteem, which is supposed to be a protective factor since low self-esteem is often associated with the prevalence of FED [42,43].

Our findings showed lower scores in both self-image and body-image among non-competitive athletes than sedentary ones. Instead, there were higher scores in both self-image and body-image among weight-dependent sports compared to weight-independent ones. These results indicate that weight-independent athletes seem to report lower levels of body and image discomfort whereas greater risk has been detected among weight-dependent athletes [15,16].

We found significant association between sport activity (all groups) and lower anxiety, depression, confusion/bewilderment, anger/hostility, fatigue/inertia states scores compared to sedentary ones. These results were also confirmed among weight-dependent as well as weight-independent athletes. These data could suggest that lower scores of mood state items among athletes may be protective in developing FED. In fact, in this study athletes seem to experience fewer states of tension and anxiety than the sedentary population, considered to be protective since symptomatic mood states (i.e., overestimation of shape, weight, and food control) are risk factors in EDs [44]. A recent large study conducted by McCabe et al. in 2019 has confirmed that depressive as well as anxiety symptoms may be included as “psychological” risk factors in a conceptual framework of risk for eating disorders [45]. Moreover, several studies have suggested that mood depression disorder (MDD) is commonly diagnosed as comorbidity with EDs [46,47,48]. According to the American Psychiatric Association, lifetime rates of MDD in individuals with EDs may vary between 50% and 75% [49], and the comorbidity of MDD with EDs has been associated with worse outcome [50,51]. Further studies showed association between MDD comorbidity with EDs and high rates of suicide attempts [52,53,54,55] and suicide-related mortality [10].

Finally, our findings showed association between sport activity and lower both self- and body-image concerns, compulsive self-monitoring, fatigue/inertia states scores compared to sedentary ones. This might add some evidence to the suggested efficacy of sport activity in improving the mood and self-confidence in general and possibly among patients with anorexia nervosa [56].

These results indicated a lower body uneasiness and weight phobia among competitive athletes in general. Instead, sedentary condition has been associated with higher scores in weight phobia (BUT WP), body image concerns (BUT BIC), compulsive self-monitoring (BUT CSM) and depersonalization (BUT D) scores, which were higher in weight-dependent athletes than weight-independent ones. In addition, the group of weight-dependent sports athletes have shown controlling behavior about their own diet (EAT-26 Oral Control), as expected. These results indicated a greater risk of FED among weight-dependent athletes.

In line with the results of previous studies [15,16], we can confirm that weight-dependent sports constituted a risk factors for FED; however, weight-dependent athletes have also been associated with protective factors such as higher self-esteem and better mood states (anxiety and depression).

According to this evidence, an FED prevention program could involve athletes at risk [57], but perhaps more importantly, an FED prevention program could be focused on both protective factors and risk factors to increase self-awareness and self-control, avoiding the demonization of weight-dependent sports. Recent prevention programs may include coaches involved in educational seminars about self-esteem, self-efficacy, mental training, sport nutrition, body composition, weight problems, and how to identify and manage disordered eating and athlete concentration problems [58]. It could also be useful to broaden the perspective on protective factors and strategies to promote health among athletes.

## 5. Limitations

This study has a number of limitations. The sample is not well balanced since we have a small group of weight-independent athletes (*n* = 26). In weight-dependent athletes, we included athletes who are more sensitive and vulnerable to change in shape and body weight that may improve their performances. Particularly, in the literature we noticed that women had greater vulnerability than men in football. In our study, football players are male only. The sample grouping in the study employed the available suggestions [15,16,29] even if there is not a well-defined classification in the literature, and this may be considered as a limitation of the study. Third, psychological assessments were done by questionnaires validated for Italian language and population, heterogeneous in age, education, and employment. However, we did not find any specific versions of these questionnaires for adolescents and adult subjects. Fourth, a total of 282 subjects, aged between 11 and 30 years were recruited for participation in this study. This could be a bias, for example in the assessment of awareness or dissatisfaction with body image or self-esteem in different ages.

## 6. Conclusions

Teen athletes seem to be a high-risk population for FED. However, our results have suggested that sport activity has shown interesting associations with protective factors even in weight-dependent sport athletes. The identification of homogeneous groups of athletes with FED predisposition can help to implement more suitable early prevention programs. Encouraging psychological factors that improve awareness of one’s body as well as promoting prevention interventions may lead to new insights on protective factors, not only risk factors, for FED in athletes. Further studies should better define the role of protective factors in preventing the onset of FED.

## Figures and Tables

**Table 1:** Differences in three groups divided by level of sport activity.

	Female (*n*)	Male (*n*)	Age (Years)	Height (m)	Weight (kg)
Competitive athletes	36	58	18 ± 4.43	1.72 ± 0.11	68.14 ± 16.25
Non-competitive athletes	36	58	20.14 ± 2.84	1.71 ± 0.10	65.37 ± 13.16
Sedentary peers	50	44	19.54 ± 4.01	1.69 ± 0.10	62.52 ± 14.76

**Table 2:** Differences in psychometric dimensions across the three groups were compared using nonparametric Kruskal–Wallis test with Dunn’s multiple comparison post-hoc testing.

	Competitive Athletes Mean ± SD	Non-Competitive Athletes Mean ± SD	Sedentary Mean ± SD	*p*
Rosenberg Self-Esteem	23.11 ± 4.89	23.04 ± 5.04	21.47 ± 4.40	**0.0177**
BSQ Self-Image	30.71 ± 15.58	23.39 ± 14.76	27.65 ± 13.82	**0.0223**
BSQ Body Image	31.90 ± 16.12	24.07 ± 15.82	28.13 ± 28.13	**0.0248**
POMS T/A	7.08 ± 5.72	6.94 ± 6.39	9.60 ± 7.46	**0.0213**
POMS D/D	5.60 ± 7.05	9.17 ± 9.30	9.23 ± 9.86	**0.0031**
POMS A/H	7.53 ± 7.53	7.60 ± 7.72	9.83 ±10.10	1.5270
POMS V/A	17.43 ± 6.87	12.90 ± 7.06	14.77 ± 5.60	**˂0.0001**
POMS C/B	5.99 ± 4.55	6.54 ± 6.17	8.78 ± 6.38	**0.0070**
POMS F/I	6.56 ± 4.03	6.18 ± 4.87	9.56 ± 6.32	**˂0.0001**
EAT-26 Dieting	2.98 ± 6.14	2.48 ± 4.75	2.28 ± 4.58	0.8549
EAT-26 Bulimia and Food Preoccupation	1.95 ± 3.08	2.59 ± 5.09	1.46 ± 1.97	0.2160
EAT-26 Oral Control	2.81 ± 3.78	2.84 ± 2.84	2.11 ± 2.89	**0.0428**
BUT WP	0.95 ± 0.89	1.08 ± 0.96	1.29 ± 1.14	0.1456
BUT BIC	0.69 ± 0.78	0.85 ± 0.84	1.16 ± 1.19	**0.0375**
BUT CSM	0.76 ± 0.63	0.87 ± 0.70	1.08 ± 0.91	**0.0362**
BUT A	0.26 ± 0.52	0.29 ± 0.49	0.41 ± 0.82	0.6838
BUT D	0.31 ± 0.52	0.40 ± 0.68	0.61 ± 0.93	0.0634
BUT GSI	0.63 ± 0.61	0.75 ± 0.66	0.97 ± 0.92	**0.0416**

BSQ Body Shape Questionnaire; POMS Profile of Mood State; T/A tension/anxiety; D/D depression/dejection; A/H anger/hostility; V/A vigor/activity; C/B confusion/bewilderment; F/I fatigue/inertia; EAT Eating Attitudes Test; BUT Body Uneasiness Test; WP weight phobia; BIC body image concerns; CSM compulsive self-monitoring; A avoidance conduct; D depersonalization; GSI global severity index. Bold numbers correspond to *p* < 0.05.

**Table 3:** Differences in psychometric dimensions across the four groups were compared using nonparametric Kruskal–Wallis test with Dunn’s multiple comparison post-hoc testing.

	Weight Dependent (*n* = 68) Mean ± SD	Weight Independent (*n* = 26) Mean ± SD	Non-Competitive (*n* = 94) Mean ± SD	Sedentary (*n* = 94) Mean ± SD	*p*
Rosenberg Self-Esteem	22.72 ± 4.68	24.12 ± 5.39	23.04 ± 5.04	21.47 ± 4.40	**0.0210**
BSQ Self-Image	30.07 ± 15.17	21.31 ± 6.00	23.39 ± 14.76	30.71 ± 15.58	**0.0005**
BSQ Body Image	30.22 ± 13.40	22.65 ± 7.00	24.07 ± 15.82	31.90 ± 16.12	**0.0015**
POMS T/A	6.87 ± 6.01	7.65 ± 4.91	6.95 ± 6.39	9.56 ± 7.46	**0.0331**
POMS D/D	5.49 ± 7.57	5.89 ± 5.60	9.17 ± 9.30	9.23 ± 9.86	**0.0056**
POMS A/H	7.44 ± 7.88	7.77 ± 6.67	7.60 ± 7.72	9.83 ± 10.10	0.5896
POMS V/A	17.79 ± 6.50	16.46 ± 7.82	12.90 ± 7.06	14.77 ± 5.60	**<0.0001**
POMS C/B	6.40 ± 4.80	4.92 ± 3.73	6.54 ± 6.17	8.78 ± 6.388	**0.0108**
POMS F/I	6.85 ± 3.62	5.81 ± 4.95	6.12 ± 4.87	9.56 ± 6.32	**0.0001**
EAT-26 Dieting	3.33 ± 5.95	2.08 ± 6.66	2.48 ± 4.75	2.28 ± 4.58	**0.0400**
EAT-26 Bulimia and Food Preoccupation	1.797 ± 2.67	2.35 ± 3.99	2.59 ± 5.09	1.46 ± 1.97	0.3818
EAT-26 Oral Control	3.02 ± 3.64	2.27 ± 4.13	2.83 ± 2.84	2.11 ± 2.89	**0.0337**
BUT WP	1.15 ± 0.92	0.40 ± 0.50	1.08 ± 0.96	1.29 ± 1.14	**0.0002**
BUT BIC	0.85 ± 0.83	0.26 ± 0.40	0.85 ± 0.84	1.159 ± 1.19	**<0.0001**
BUT CSM	0.89 ± 0.66	0.40 ± 0,37	0.87 ± 0.70	1.08 ± 0.91	**0.0004**
BUT A	0.32 ± 0.58	0.13 ± 0.26	0.29 ± 0.49	0.41 ± 0.81	0.4650
BUT D	0.39 ± 0.57	0.12 ± 0.24	0.40 ± 0.68	0.61 ± 0.93	**0.0052**
BUT GSI	0.77 ± 0.64	0.27 ± 0.28	0.75 ± 0.66	0.97 ± 0.02	**<0.0001**

BSQ Body Shape Questionnaire; POMS Profile of Mood State; T/A tension/anxiety; D/D depression/dejection; A/H anger/hostility; V/A vigor/activity; C/B confusion/bewilderment; F/I fatigue/inertia; EAT Eating Attitudes Test; BUT Body Uneasiness Test; WP weight phobia; BIC body image concerns; CSM compulsive self-monitoring; A avoidance conduct; D depersonalization; GSI global severity index. Bold numbers correspond to *p* < 0.05.
